# Schistosomiasis Chemotherapy, Chemoprevention, and Vaccines: History, Progress, and Priorities

**DOI:** 10.1002/iid3.70054

**Published:** 2024-11-19

**Authors:** Alaa Oqalaa E. Alibrahim, Walaa A. Elkholy, Mona M. El‐Derbawy, Noha F. Zahran, Athanasios Alexiou, Marios Papadakis, Gaber El‐Saber Batiha

**Affiliations:** ^1^ Department of Internal Medicine College of Medicine, Jouf University Sakaka Saudi Arabia; ^2^ Department of Parasitology Faculty of Medicine for Girls, Al‐Azhar University Cairo Egypt; ^3^ Department of Parasitology Faculty of Medicine for Girls, Al‐Azhar University New Damietta City Egypt; ^4^ University Centre for Research & Development Chandigarh University Mohali Punjab India; ^5^ Department of Research & Development Funogen Athens Greece; ^6^ Department of Surgery II University Hospital Witten‐Herdecke, University of Witten‐Herdecke Wuppertal Germany; ^7^ Department of Pharmacology and Therapeutics Faculty of Veterinary Medicine, Damanhour University Damanhour AlBeheira Egypt

**Keywords:** chemoprophylaxis, praziquantel, schistosomiasis, vaccines

## Abstract

**Background:**

Schistosomiasis is a major human disease of public health importance. Freshwater snails serving as intermediary hosts and human interaction with surface water tainted by feces or urine are both necessary components of the transmission cycle. *Schistosoma haematobium*, *Schistosoma mansoni*, and *Schistosoma japonicum* are the primary pathogen species. Over 250 million individuals are infected globally, according to the World Health Organization, causing significant morbidity and an estimated loss of 1.9 million disability‐adjusted life years, a number that is probably underestimated. Immunological protection is slowly built up through complex immunological systems, although innate factors also play a role. Chronic schistosomiasis affects mainly individuals residing in poor rural area. Vaccination is considered as one of the most sustainable options for the control of any pathogen, but schistosomiasis vaccine for humans or animals is not available till now despite the discovery of numerous potentially promising schistosome vaccine antigens.

**Objective:**

To provide an overview of the schistosomiasis chemotherapy, chemoprevention, and vaccines history and progress.

**Design:**

Review article.

**Data Sources:**

PubMed, ISI Web of Science, Science Direct, and the World Health Organization database.

**Conclusion:**

Favorably praziquantel (PZQ) is a medication with excellent chemopreventive treatment compliance. Due to the extensive usage of PZQ, there is a great deal of debate surrounding the emergence of drug resistance. PZQ is effective against all species of schistosomes, schistosomiasis prevalence has remained largely unaffected, due to reinfection in high transmission areas and growing juvenile worms that were not affected by the drug, even though the need for a schistosomiasis vaccine is even more pressing.

## Graphical abstract

Schistosomiasis is a major human disease of public health importance. The goal is to provide an overview of the history and advancements in schistosomiasis chemotherapy, chemoprevention, and vaccine research. Praziquantel is effective against all species of schistosomiasis, but schistosomiasis prevalence has largely remained unaffected, due to reinfection in high transmission areas and growing juvenile worms that were not affected by the drug, even though the need for a schistosomiasis vaccine is made even more pressing.

## 1 Introduction

Schistosomiasis, a neglected tropical illness caused by blood flukes of the genus *Schistosoma*, continues to be a disaster for humanity. It is a waterborne illness that has a significant impact on communities in South America, Asia, and Africa. Apart from some animal schistosomes *Schistosoma haematobium, Schistosoma mansoni*, and *Schistosoma japonicum* can cause human infection [1]. It is a serious condition with significant public health implications in people that affects more than 70 tropical and subtropical nations and accounts for over 70 million Disability Adjusted Life Years (DALYs). Two hundred and fifty‐two million people in the tropics and subtropics are affected by it [2, 3, 4]. After hookworm, it is the second most frequent Neglected Tropical Diseases (NTD). The illnesses affect more than 78 nations and nearly 800 million people are exposed to the diseases. It is a major source of illness and mortality in Africa, South America, the Caribbean, the Middle East, and Asia. It is the third most serious tropical disease worldwide [4].

One of the efforts by Cawston in 1918, attempted to determine the methods of transmission of *S. haematobium* in Natal, South Africa, forms the historical context that provides the bases to assess our progress.

**Figure 1:**
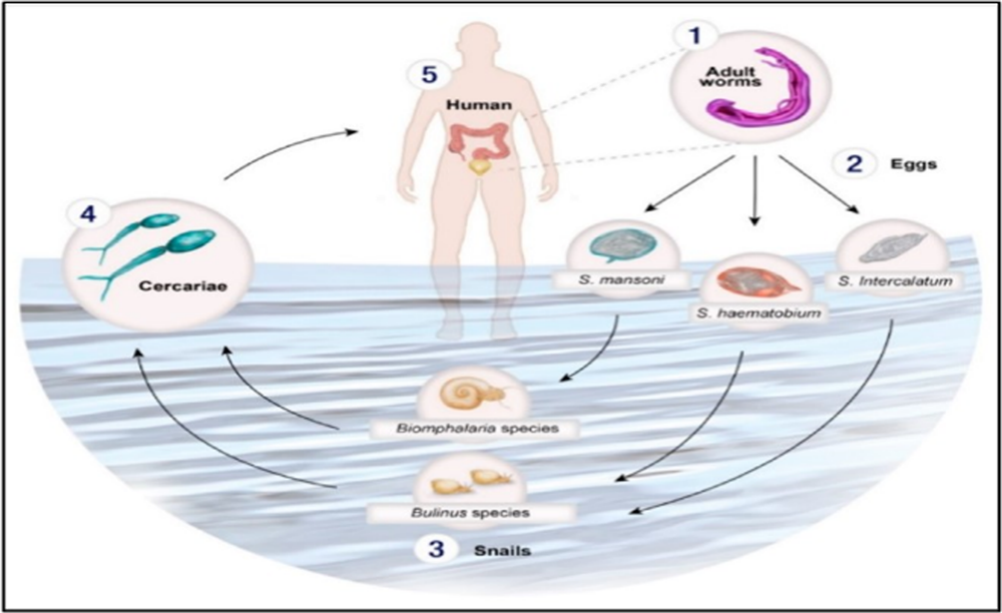
Schistosome life cycle [6].

The schistosome life cycle is complex, with morphologically distinct stages occupying several ecological niches. Infective cercariae swim in fresh water to find and infect a mammalian host. After host invasion, cercariae transform into schistosomula, and adapt to survival in the host bloodstream. The schistosomula mature into adult male or female schistosomes, which pair and produce eggs, eggs are excreted from the host. In fresh water, the eggs hatch into miracidia, which infect a snail host and develop into sporocysts. Daughter sporocysts generate infectious cercariae, completing the life cycle. The cercaria is transiently free living and represents the first interaction point in the parasite life cycle with the human host (Figure 1) [5].

The complicated relationship of schistosome species and their snail hosts has been better defined by molecular biology techniques, but they have also revealed the extent to which human and animal schistosomes have hybridized, which may have an impact on the adaptability of the parasites [7]. Microarrays, RNA‐seq, and proteomics have all been utilized to study this cercarial stage; however, there is still much to learn about the transcriptional and translational regulatory mechanisms at this level [5].

The infection site, the infecting species, and the parasite burden all affect how the infection clinically manifests. Detecting eggs in urine or feces after concentration is a traditional method for diagnosing this illness, although there are additional serological tests and molecular assays available [1]. It is a poverty associated disease, therefore taking preventative steps like having access to clean water and sanitary facilities can help lower the chance of infection. The only effective preventative measures in the absence of a vaccine are the eradication of snails and the control of water pollution, both of which are frequently absent. The World Health Organization (WHO) predicted that by 2010, at least 75% of all schoolchildren who were at risk of schistosomiasis morbidity would have received treatment with the aim of reversing morbidity. It is acknowledged the significance of current global projects like the Schistosomiasis Control Initiative (SCI) operating in Mali, Niger, Burkina Faso, Zambia, Tanzania, Kenya, and Uganda. Since this significantly lowers the cost of medication administration, there are advantages to integrating the control of schistosomiasis with other disease control programs, such as those for gastrointestinal helminths and/or lymphatic filariasis (LF). For such programs to be successful, they must be lasting. On the periphery of the schistosomiasis distribution, nations have either completely eradicated the illness or have made headway in doing so. Therefore, it will be crucial for Ministries of Health and Education to budget for the prevention and treatment of diseases of poverty with a focus on the African continent, in addition to school health, and to use money from a variety of sources, including government funds, pooled donor contributions, or bilateral and international agencies [1, 2, 4].

The new WHO Roadmap for NTDs aims to eradicate schistosomiasis as a public health burden globally. It is one of the top 10 vaccinations that must be produced immediately to enhance worldwide public health. The WHO has redoubled its efforts to combat NTDs by recently modifying its NTDs 2021−2030 strategy to accomplish the Sustainable Development Goals (SDGs) [8]. Controlling NTDs requires a multidisciplinary and integrated strategy that includes studying the environment, route of transmission, disease immunology, inflammation and enhancing access to diagnosis, treatment, and vaccine development [9]. Vaccination is regarded as one of the most sustainable methods of disease management, however despite the identification of multiple potentially effective schistosome vaccine antigens, no schistosomiasis vaccine for humans or animals is currently available [10].

The review aims to provide an overview of the schistosomiasis chemotherapy, chemoprevention, and vaccine history and progress.

## 2 Methods

### 2.1 Design: Review Article

**Figure 2:**
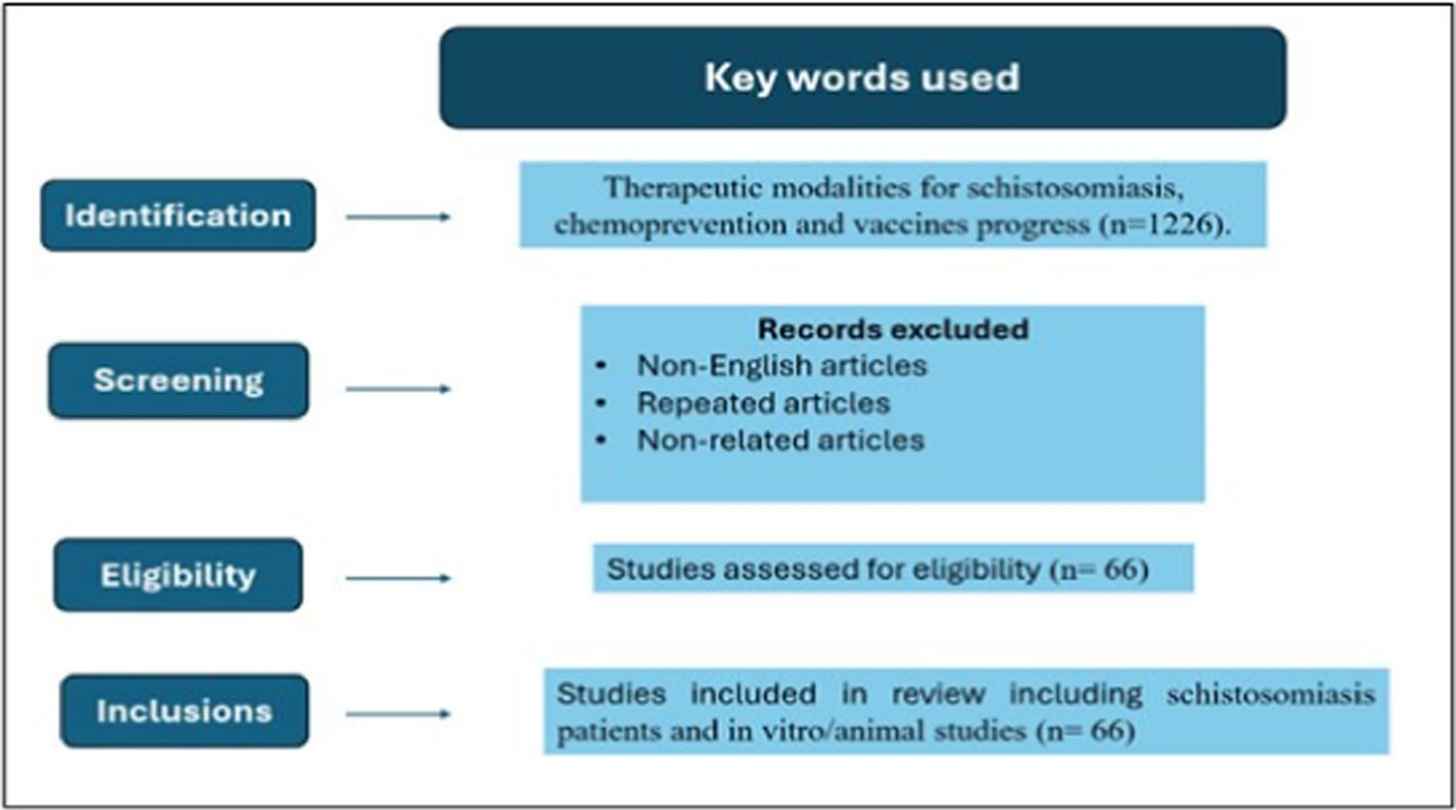
Study flowchart.

The current review covered materials such as literature reviews, systematic reviews, and clinical trials. A search strategy was devised using medical subject headings (MeSH) to search the PubMed, MEDLINE scientific databases, ISI Web of Science, Science Direct, and the WHO database. The MeSH terms utilized included schistosomiasis, chemotherapy, therapeutic modalities, chemoprevention, and vaccines progress. The total number of papers included or used for the review nearly 66 articles. The inclusion criteria encompassed studies published in the English language from 1996 to 2023, with a particular emphasis on the most recent literature pertaining to different therapeutic modalities for schistosomiasis (Table 1, Figure 2).

**Table 1:** Inclusion criteria for search strategy.

Parameter	Criterion
Trials and studies	Studies published in the English language from 1996 to 2023, especially the most recent literature pertaining to different therapeutic modalities for schistosomiasis.
Intervention	Studies related to usage of praziquantel as chemoprevention or therapeutic agent. Studies related to schistosomiasis vaccines progress.
Outcome	Remission or control of *Schistosoma* infection.
Setting	All settings

## 3 Results

### 3.1 Praziquantel (PZQ)

PZQ is the medicine of choice today for treating all kinds of schistosomiasis [1, 4]. Given that there isn't an effective vaccination for the condition, favorably PZQ is a medication with excellent chemopreventive treatment compliance. Since 1982, PZQ has been used to treat infected patients, sparking a revolution that directly influenced bulk medication distribution initiatives. PZQ is used widely as part of the schistosomiasis treatment. It is curative therapy and can be administered several times to completely eradicate the infection. According to the WHO, PZQ is administered at the recommended single oral dose of 40 mg/kg body weight in endemic regions as apart from mass drug administration (MDA) programs [11]. With the help of the Bill & Melinda Gates Foundation and a significant drop in cost, PZQ is now more frequently used in sub‐Saharan Africa [4, 7, 12]. Though these campaigns reduced worm burdens have lessened the serious pathological effects of high worm burdens (such as the squamous cell carcinoma associated with *S. haematobium* infections), their efficacy was compromised, and incomplete recovery due to PZQ's ineffectiveness against immature juvenile flukes and reinfection was observed [7, 11]. However, due to its poor solubility (hydrophobic nature) after oral intake which leads to its slow absorption from the gut lumen with low bioavailability and it was subsequently observed that PZQ failed to provide complete recovery in some treated populations [13]. Notably, after being absorbed from the GIT, PZQ undergoes fast metabolism in the liver, resulting in a short half‐life in the bloodstream (first‐pass hepatic metabolism) [14]. This reduces its effectiveness against *S. mansoni* juveniles in the systemic circulation with inadequate drug exposure, and the incidence of reinfection has been documented [15].

Due to the extensive usage of PZQ, there is a great deal of debate surrounding the emergence of drug resistance. PZQ drug resistance has been demonstrated in a lab strain. The gene for resistance has been identified [16]. On the contrary, a systematic review by Abaza [17] spotlighted and delineated the factors leading to reduced PZQ effect on schistosomiasis. The authors clarified that host cytochrome P450 (CYP) enzyme is to blame for the lower medication efficacy through the host CYP genetic variation that results in individual variations in PZQ metabolism, rather than extended or widespread usage of PZQ. According to some theories, CYP either mediates PZQ metabolism directly or indirectly through drug−drug interactions by way of an enzyme process that interferes with PZQ activity [18]. So, the creation of novel techniques that can improve PZQ‐based chemotherapy is urgently needed [12]. Drug delivery methods utilizing lipid‐based nanocarriers, such as silica nanoparticles, in conjunction with PZQ [19], liposomes [20], solid lipid nanoparticles [21], and noisome [22] were shown to increase PZQ bioavailability and anti‐schistosomal activity.

Mohamed et al. [23] observed promising additive effect of ursodeoxycholic acid (UDCA) as a cholagogue when used with PZQ in the treatment of schistosomiasis mansoni and hypothesized that the combination of UDCA decreased worm burden in the liver and the mesenteric vessels. The mean total count of ova in the tissues of infected mice, the mean granuloma diameter, and granuloma numbers in the infected animals' livers also decreased.

Kim et al. [24] reported that the function of UDCA after 8 weeks of therapy, this cholagogue improved liver function via the phenylalanine/tyrosine pathway microbiome remodeling in patients with liver disease by decreasing *Lactobacillus* and *Bifidobacterium* numbers. *Lactobacillus*, *Bifidobacterium*, *Bacteroides*, and *Clostridium*‐rich microbiomes were found to interfere with gut bacterial metabolite synthesis. UDCA was also characterized as a hydrophilic nontoxic bile acid produced in the liver and eliminated in human bile. It is a cytoprotective, antiapoptotic, membrane stabilizing, and antioxidative immunomodulatory substance. As a result, it is prescribed to individuals with both cholestasis and non‐cholestasis liver disorders. However, with the likelihood of PZQ resistance developing, the possible involvement of alternative medications, such as artemether, in the management of schistosomiasis is being investigated [2, 4].

Consistent with the disruptive mechanism of action of PZQ, following PZQ treatment could enhance antigen recognition of antigen‐protein‐isoforms because of antigenic unmasking. Compared to pretreatment sera of *S. haematobium* patients', posttreatment serum samples showed a greater number of proteins. Moreover, some proteins and their isoforms are recognized only in posttreatment serum samples [25].

The proteins showed enhanced recognition post treatment are mainly associated with the proteins of parasite musculature and glycolytic metabolism [26]. This increased antigen recognition enhanced better neutralization by antibodies induced by vaccination also increased affinity maturation of immunoglobulin responses such as worm specific IgE [27], an effect that encourge to co‐administered vaccines and moreover lead to a more sustained antibody response following vaccination [28]. Following PZQ chemotherapy, IgE to egg antigens are boosted besides antibody titers IgG1, IgG2, IgG4, IgM against worm antigens and IgG2. Indeed, PZQ therapy significantly modifies the polarization and amount of schistosome‐specific cytokine responses with long‐term immunological effects. Following PZQ treatment, a Th2 biassed response is shown in the human host as evidenced by elevated levels of parasite‐specific IgE, eosinophil counts, and soluble‐high and low affinity IgE receptors on eosinophils and CD23 + B cells [29, 30], that related to reinfection resistance [31]. Furthermore, PZQ treatment reduces immuno‐suppressive T‐reg, Th17 cell counts, and IL‐10 cytokine levels to lessen the immune suppression brought on by schistosomes [29]. Following PZQ treatment, infection clearance leads to a rise in effector T cell frequency, an increase in the cytokine response specific to schistosomes, and a drop in T reg levels. This is in contrast to the immune‐suppressive profile that is present during infection, which includes increased numbers of CD4 + CD25 + FOXP3 + T cells, antigen‐specific hyporesponsiveness, and distortion of the T cell memory pool [32].

**Figure 3:**
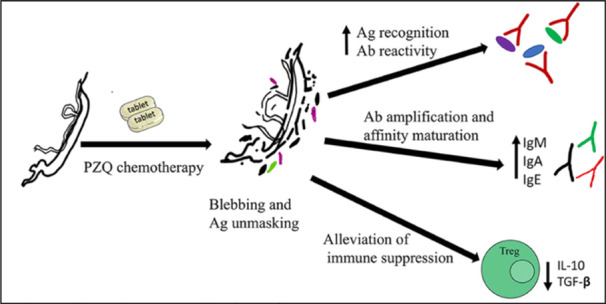
Summarized scheme of PZQ‐induced immune‐modulatory responses that potentially affect vaccine response upon co‐administration/delivery. PZQ acts on parasite tegument causing vacuolation and blebbing. This unmasks worm antigens and elicits an increased antigen recognition, antibody reactivity, amplification and affinity maturation, and alleviates the immune suppression induced by live parasites [34]. Ab, antibody; Ag, antigen; PZQ, praziquantel.

In conclusion, PZQ‐induced antigenic release elicits both short‐ and long‐term immune modulatory effects, despite the fact that PZQ has been shown to be nonimmunogenic in and of itself, meaning that it has no influence on vaccination effectiveness this might thus enhance or conceal vaccination reactions (Figure 3) [33].

This is especially crucial since adult individuals in endemic regions for future schistosome vaccination studies will have immune profiles changed by repeated exposures and/or PZQ therapy. Therefore, PZQ‐vaccine co‐delivery may either be a highly useful, more comprehensive approach to improve vaccination response and effectiveness or it may hinder the ability of late‐stage clinical studies to identify vaccine efficacy. Thus, before implementing such tactics in Phase 3 research, several vaccination regimens with PZQ chemotherapy need to be evaluated to determine the most suitable vaccine‐PZQ combination and immunization‐chemotherapy treatment plan [34].

### 3.2 Vaccines Against Schistosomiasis

The need for a schistosomiasis vaccine is made even more pressing by the fact that, even though PZQ is effective against all species of schistosomes, schistosomiasis prevalence has remained largely unaffected, due to reinfection in high transmission areas and growing juvenile worms that were not affected by the drug, both of which restore the main levels of infection after treatment [35].

Broad use of PZQ may result in the emergence of drug resistance in the future. Because schistosomes do not multiply within the human host (or other important vertebrate hosts), there is no need for a vaccine to be 100% effective, and mathematical modeling reported that even a partly protective vaccine would play a role in reducing schistosome infections and hindering transmission [36].

According to the schistosome life cycle, the break in the worms' protective armor that would be most desirable for targeting with a vaccine in humans is the short interval between cercarial skin penetration and the presence of schistosomula in the lungs, when the parasite should be most vulnerable to immune attack as it adapts to the definitive host schistosome infections and disrupts transmission [37].

Targeting this early stage of the parasite in humans is especially intriguing because there are no immunopathological problems associated with the eggs released by mature worms. The first human vaccination experiment was against *S. haematobium* (rSh28GST, aka Bilhvax), which terminated with poor results due to insufficient efficacy [38].

Sm‐TSP‐2 (*S. mansoni* tetraspanin 2), Sm‐p80 (*S. mansoni* calpain), and Sm14 are three more vaccine candidates in early Phase 1/2 studies, with preliminary data not yet available [39]. For Sm‐p80, there are data for baboons. When compared to unvaccinated control animals, the vaccination demonstrated strong preventive effectiveness against the spread of *S. mansoni* infection and was linked to noticeably reduced egg‐induced pathology. The vaccine's ability to prevent parasite transmission is shown by a 35‐fold decrease in fecal egg excretion in vaccinated animals and an 81.51% reduction in egg hatching into the snail‐infective stage (miracidia). Significantly increased Sm‐p80 expression in female worms and Sm‐p80‐specific antibodies in baboons that have received vaccinations seem to be key components of vaccine‐mediated protection [40].

The zoonotic nature of *S. japonicum* complicates transmission since cattle are the principal reservoir hosts and are responsible for about 90% of parasite egg contamination in the environment [41, 42]. In the context of *S. japonicum*, therefore, vaccination of bovines is required as part of a strategy known as “transmission blocking vaccine” since it would help in the long‐term prevention of human (and animal) infection [43].

Across a wide range of experimental animal models, the radiation‐attenuated (RA) cercariae vaccination continually provides protection against schistosomiasis [44]. However, for practical and ethical reasons, the RA vaccine cannot currently be used in humans. However, advancements in systems biology and catalogues of highly immune sera from earlier animal vaccination experiments provide a chance to discover highly immunogenic proteins from schistosomes induced by the RA vaccine. Sera from *S. mansoni* RA vaccinated mice and IFN‐γ receptor mutant mice were recently utilized to identify peptides from over 40 proteins that might be prioritized for further study in multi epitope vaccine constructions [45]. Furthermore, the availability of new adjuvants that may selectively employ immune responses, as well as advancements in immunological research, allow for cell signaling studies to determine the reactions that each vaccination must elicit [46].

To achieve significant improvements in schistosomiasis control would require a variety of tactics, and a vaccination against schistosomes is one of those tools that would fill the gap left by the immediate benefits of MDA efforts [6].

### 3.3 Human Behavior, Cost‐Effectiveness Analysis, & Development Priorities of a Schistosomiasis Vaccine

In the health sector, cost‐effectiveness analysis is often used as a tool to decide where to allocate uncommon resources for disease prevention or control. The validity of the behavioral hypotheses that supported the economic analysis specifically is rarely considered. It is possible to decide which research projects should be emphasized using cost‐effectiveness analysis to develop an effective vaccine for schistosomiasis. Researchers need a vaccine with sufficient duration to provide protection to be administered as part of the routine immunization program of children to guarantee that the vaccine would be more cost‐effective than the currently preferred option for the control of schistosomiasis, chemotherapy based on PZQ. A round of chemotherapy should only cost US$4.30 per child more than the expense of adding it to the current vaccinations schedule. Ideal effectiveness would be more than 80%. However, a few cultural and behavioral elements that are often disregarded in cost‐effectiveness research are what determine these outcomes. Low rates of school attendance would make contacting children for a treatment program more expensive and would also make vaccines seem more appealing. Children must also follow instructions carefully each year for several years for chemotherapy to be successful. Chemotherapy's efficacy would decline as time passed as compliance rates dropped, making vaccines more appealing. The success of the whole childhood immunization program may be challenged if mothers believed the vaccine to be useless and refused to send their children for vaccination, even if a vaccine may still be more affordable than chemotherapy at relatively low levels of vaccine effectiveness [47].

### 3.4 Progress of *Schistosoma* Vaccines

An approach for the development of a vaccine against *S. mansoni* has been determined, focusing on six priority recombinant antigens. An Asian *S. japonicum* vaccine development has also been established, initially to develop a vaccine to block transmission in cattle and oxen, important reservoirs of the disease in Asia, and ultimately a human vaccine. These vaccines will be used in disease‐endemic tropical countries, so optimal stability will be of chief importance [48].

Experimental animal models that review schistosomiasis immunology, disease progression, and pathology observed in humans are important in testing and validation of control interventions. Nonmammalian intermediate hosts and other vertebrates promote transmission of schistosomiasis and have been used as experimental model systems. Antibody assays are appreciated tools for evaluating efficacy of candidate vaccines, and sera from graded infection experiments are useful for evaluating diagnostic sensitivity of different targets [12].

The target population's previous exposure to infections and schistosomes may have an impact on the effectiveness and responsiveness of the vaccination, and exposure in utero may increase the variability of vaccine responses, which calls for careful consideration. The process of choosing appropriate trial populations becomes more difficult due to differences in acquired immunity development, age‐dependent exposure distribution, and immunological profile. In populations with persistent infections, schistosomes have the capacity to cause immunological suppression mediated by T‐reg/IL‐10 [34].

Schistosomiasis vaccines planners targets that there is urgent need for the formation of an effective anti‐schistosome vaccine pipeline that makes use of cutting‐edge technology (including developing mRNA vaccines and exploiting CRISPR‐based technologies) to help future vaccine discovery, design, manufacture, and deployment. Novel research tools such as sequencing, improved understanding of disease pathogenesis and utilization of experimental models to assist with evaluating performance of new approaches [48].

### 3.5 Different Parts of *Schistosoma* as a Component of a Schistosomiasis Vaccine

#### 3.5.1 Lipid Core Peptide (LCP) Targeting the Cathepsin D Hemoglobinase of *S. mansoni* as a Component of a Schistosomiasis Vaccine

The self‐adjuvating LCP system offers a safe alternative vaccine delivery strategy, abolishing the need for additional adjuvants such as CpG Alum. LCP acts as a scaffold for an epitope located on the surface of the cathepsin D hemoglobinase (Sm‐CatD) of the human blood fluke *S. mansoni*. Sm‐CatD plays a crucial role in digestion of the fluke's blood meal and has been revealed to be efficient as a subunit vaccine in a murine model of human schistosomiasis. Some of the peptides were fused to a self‐adjuvating lipid core scaffold to generate LCPs. Mice were vaccinated with unadjuvanted peptides, peptides formulated with Freund's adjuvants, or LCPs. Antibodies generated to LCPs recognized native SmCatD within a soluble adult schistosome extract, and approximately totally abolished its enzymatic activity in vitro. Using immunohistochemistry, anti‐LCP antibodies bound to the native Sm‐CatD protein in the esophagus and anterior regions of the gastrodermis of adult flukes. Vaccines offer an alternative control strategy in the fight against schistosomiasis, and so more advances of LCPs containing multiple epitopes from this, and other vaccine antigens should become a research priority [49].

#### 3.5.2 Schistosome Membrane Proteins as Vaccines

The outermost surface of intra‐mammalian stages of the parasite, the tegument, is the key to the parasite's accomplishment and the most susceptible target for vaccines and drugs. Over the past 2 years the proteome of the *S. mansoni* tegument has been investigated and these studies revealed unexpectedly few proteins that are predicted to be accessible to the host immune response, namely proteins with at least one membrane‐spanning domain. In particular, the tetraspanin family of integral membrane proteins appears to be profusely represented in the tegument, and convergent data consuming the mouse vaccine model and correlates of protective immunity in naturally exposed people suggests that this family of membrane proteins offer great promise for schistosomiasis vaccines. Through the recent improvements in schistosome genomics and proteomics, a new set of potential vaccine antigens is presented and these permit thorough investigation and proper funding over the next few years [50].

#### 3.5.3 Role of Protein Kinases (PKs) Against *S. japonicum*

*The S. japonicum* infection is chiefly dominant in Asia. PKs are enzymes that catalyze the phosphorylation of proteins and can contribute in many signaling pathways in vivo. Recent studies confirmed the essential roles of PKs in the growth and development of *S. japonicum*, together with schistosome−host interactions, and research have screened drug targets about PKs from *S. japonicum* (SjPKs), which provide new chances of developing new treatments for schistosomiasis [51].

#### 3.5.4 Expression, Purification, and Human Antibody Response to a 67 kDa Vaccine Candidate for Schistosomiasis Japonica

*S. japonicum* surface membrane protein 67 (SJ 67) is homologous to a family of actin‐binding proteins. It is known by a mouse monoclonal antibody (mAb 6) that confers resistance to challenge infection in passive transfer experiments. Purified recombinant rSJ67 had a molecular weight of 67 kDa and N‐terminal sequencing confirmed that the first five amino acids of the recombinant protein matched the predicted sequence for the SJ67 gene. In western blot analysis, rSJ67 was known as the SJ67 specific mAb 6 antibody. IgG antibodies in sera from schistosomiasis infected volunteers living in an endemic area of the Philippines (*n* = 13) recognized rSJ67 with 4.7‐fold greater median fluorescence compared to uninfected North American controls (*n* = 5) (*p* < 0.009). Together, these data confirmed the expression and purification of recombinant SJ67 and its immuno‐reactivity with sera from *S. japonicum* infected humans [52].

### 3.6 Different Antigens of *Schistosoma* as Adjuvants With Other Vaccine

#### 3.6.1 *S. mansoni* Soluble Egg Antigens (SEA) Enhance *Listeria monocytogenes* Vector HIV‐1 Vaccine Induction of Cytotoxic T Cells

Many vaccines incorporate adjuvants to stimulate the recipient's immune system and enhance vaccine‐specific responses. While vaccine development has been improved from attenuated organisms to recombinant protein or use of plasmid DNA, the development of new adjuvants that increase immune responses has not taken a step. Prior studies have shown that the complex mixture of molecules that comprise saline SEA from *S. mansoni*eggs functions to promote CD4(+) T helper 2 (Th2) responses. Therefore, the coadministration of SEA with a Listeria vector HIV‐1 Gag (Lm‐Gag) vaccine would suppress host cytotoxic T lymphocyte (CTL) and T helper 1 (Th1) responses to HIV‐1 Gag epitopes. Amazingly, instead of driving HIV‐1 Gag‐specific responses toward Th2 type, that coadministration of SEA with Lm‐Gag vaccine significantly increased the frequency of gamma interferon (IFN‐γ)‐producing Gag‐specific Th1 and CTL responses over that seen in mice administered Lm‐Gag only. Analysis of the functionality and durability of vaccine responses proposed that SEA not only enlarged different memory T cell compartments but induced functional and long‐lasting vaccine‐specific responses as well. These results suggest there are components in SEA that can synergize with potent inducers of strong and durable Th1‐type responses such as those to Listeria. We assume that SEA contains fractions that, if defined, can be used to expand type 1 proinflammatory responses for use in vaccines [53].

#### 3.6.2 *S. mansoni* SEA and Recombinant Omega‐1 Modulate Induced CD4^+^ T‐Lymphocyte Responses and HIV‐1 Infection In Vitro

Parasitic helminths have advanced several strategies to evade human immune responses. Such effects will likely have consequences for HIV‐1 transmission and disease progression. SEA, through kappa‐5, can powerfully block dendritic cells DC‐SIGN mediated HIV‐1 trans‐infection of CD4^*+*^ T‐lymphocytes, nevertheless not block cis‐infection. DC exposed to SEA during maturation under Th2 skewing conditions, induce T‐cell populations that are less susceptible to HIV‐1 R5 infection compared to cells induced by unexposed DCs. This limited infection profile was not associated with down‐modulation of CCR5 surface expression or else stated differences in cytokine/chemokine production. Using recombinant omega‐1, an abundant component of SEA, HIV‐1 R5 infection was similarly inhibited with no effect on HIV‐1 ×4 infection levels. Consequently, SEA possesses antigens, namely omega‐1, that can modulate HIV‐1 infection and possibly influence disease course in co‐infected individuals [54].

#### 3.6.3 Potential Benefits of Reduced Prevalence of Helminths on Immunity and Vaccination Against Other Diseases

Chronic parasitic helminth infections can be detected by the production of T helper type 2 (Th2) cells, which establish a Th2 cytokine profile and split the immune system into a systemic Th2 bias Maizels and McSorley [55]. Animal research employing knockout transgenic mice indicated that helminth parasites polarize Th2 via the cytokines IL‐4 and IL13 via the IL‐4 receptor alpha (IL‐4Ra) signaling mechanism. During *S. mansoni* infection, IL‐4 regulates the production of the transcription factor Foxp3 in regulatory T cells via the IL‐4Ra signaling mechanism [56]. T reg cells serve an important role in immune system control by maintaining self‐tolerance and inhibiting excessive immunological responses that are detrimental to the host. The overall outcome is the generation of a Th1/Th2 balance that is skewed toward a Th2 bias, which is manifested as hypo‐responsiveness to bystander antigens through generalized immune regulation. Naturally, Th1 and Th2 responses are antagonistic, and a high Th2 environment tends to diminish Th1 cytokine responses, indicating the possibility for persistent helminthic infections to lower Th1 immune responses. Strong and polyfunctional Th1 responses are critical in activating protective immune responses against several diseases including as tuberculosis, malaria, HIV‐1, and SARS‐CoV‐2. antigens. As a result, chronic parasitic helminth infections have the potential to exacerbate the pathological consequences of certain infections, enable secondary infections, and suppress vaccine‐specific immune responses, reducing vaccine immunogenicity and protective potential infections such as tuberculosis, malaria, HIV‐1, and SARS‐CoV‐2 [57].

There is growing data from preclinical and clinical research that synchronized helminthiasis suppresses vaccination responses. Two African investigations indicated that *S. mansoni* infections reduced anti‐measles antibodies in already vaccinated schoolchildren while producing a quicker drop in antibody levels to hepatitis B and tetanus toxoid vaccinations over time [58].

Infected mice with *S. mansoni* exhibit downregulated HIV‐1‐specific immune responses, which results in a delay in the vaccinia virus's clearance from the liver, spleen, and lungs. As S. *mansoni*‐infected mice responded poorly to a prospective HIV‐1 DNA vaccine, the eradication of helminth infection restored vaccine‐specific IFN‐γ responses in *S. mansoni*‐infected animals. In the mouse and baboon models, chronic *S. mansoni* infection reduces the ability of first‐generation candidate HIV vaccines, SAAVI DNA‐C2 and SAAVI MVA‐C, to induce and strengthen the immune system. In addition, after vaccination with a clinical human papillomavirus (HPV) vaccine, the levels of anti‐HPV antibodies in the sera of *S. mansoni*‐infected baboons were lower than those in sera from *S. mansoni*‐free animals. In humans, the development of the immune system in the fetus is characterized as infection‐free until delivery, and the mother's antibodies are largely transferred across the placenta, boosting the child's immunity in the early years of life. However, recent research has shown that maternal illnesses may expose infants to antigens in utero, changing the situation. According to studies, parasite antigens are sensitized in neonates of women who have helminth illnesses such schistosomiasis and filariasis, which are characterized by the production of IL‐4, IL‐5, IgE, and IFN‐ γ [59].

According to recent research, in utero sensitization triggers immunologic memory that lasts throughout infancy. Additionally, a suppressed immunological status characterized by the growth of Treg cells and IL‐10 has been linked to maternal schistosomiasis. Active suppression caused by IL‐10 and other regulatory mechanisms may guard against the emergence of severe illness as well as reduced responses to critical immune components implicated with vaccination effectiveness. In addition to being positively correlated with schistosome‐induced IL‐10 levels, maternal schistosome infection has been linked to reduced protective IgG antibody formation to the *Hemophilus influenzae* type b (Hib) and diphtheria toxoid (DT) vaccinations. Additionally, among preschoolers, decreased IgG levels to the measles vaccination and suppression of BCG‐specific IFN‐ γ, which are linked to protection, have been documented [60].

According to some research, these effects may be mitigated or avoided by providing antiparasitic medication to prevent immunomodulation. This was supported by studies by Noah, who found no connection between maternal infection and decreased effectiveness of children vaccines. Unlike other research that found maternal malaria, hookworms, or schistosomiasis infection to be linked to decreased *Streptococcus pneumoniae* antibody levels in the newborns. According to other research, children who had previously been infected with *S. mansoni* and had PZQ therapy had greater anti‐measles IgG levels than untreated children. These studies emphasized the necessity of control and prevention of parasitic infections during pregnancy [5].

In endemic areas the helminth infections also helped to reduce prevalence of allergic diseases. The fetomaternal interface is altered in various ways specifically as a result of maternal helminth infection, and these changes in turn affect how offspring respond to allergens. In research that inspected the impact of pregnancy on progress from Th1 to Th2 and final regulatory state in rodent model of schistosomiasis, delineated that offspring from mothers in Th1 and regulatory state were guarded against allergic airway inflammation (AAI) [61] (Table 2).

**Table 2:** Summary of the main key points studied in different articles.

Authors	Year of publication	Objectives, key findings
Wilson et al., Abaza et al., Zdesenko and Mutapi	2020, 2021	Praziquantel is the primary treatment for schistosomiasis
Tawfeek et al.	2019	Lipid‐based nanocarriers for PZQ
Mohamed et al.	2023	PZQ with other treatments like ursodeoxycholic acid
Cohen et al.	2016	The need for a schistosomiasis vaccine is urgent
Kumar et al.	2017	New vaccine formulations
Naglaa et al.	2016	Optimal combination of PZQ and vaccines in treating schistosomiasis
Leonardo et al.	2019	Success of vaccination programs
Driciru et al.	2021	Schistosomes can cause immunological suppression, particularly in populations with persistent infections, which may affect vaccine outcomes
Molehin et al.	2022	Recombinant antigens and blocking transmission in vaccine development
Hotez, Riveau	2019, 2018	Human & cattle vaccine trials
Mouser et al.	2019	Lipid core peptide (LCP) targeting Sm‐CatD as a vaccine candidate
Dougall et al.	2014	Schistosome membrane proteins as a vaccine candidate
Jolly et al.	2007	PKs offers new avenues for developing treatments and vaccines against schistosomiasis
Loukas et al.	2007	SJ67 as a vaccine candidate for *S. japonicum
Evans and Guyat	1997	Schistosome soluble egg antigens as adjuvants
Li et al.	2021	Complex interaction between helminth infections and immunity
Maizels et al.	2016	Chronic helminth infections, such as schistosomiasis, can skew the immune system toward a Th2 bias
Cohen et al.	2016	Schistosomiasis vaccine as one of the top 10 vaccines in need of urgent research
Molehin et al.	2022	Urgent need for an effective vaccine pipeline that leverages cutting‐edge technologies like mRNA vaccines and CRISPR

## 4 Discussion

PZQ is the primary treatment for schistosomiasis, a disease without an effective vaccine. Although PZQ is effective against all schistosome species, the drug efficacy can be limited due to issues such as poor solubility, rapid metabolism, low bioavailability, reinfection in high‐transmission areas, and the drug's ineffectiveness against juvenile worms. This can restore infection levels after treatment, and broad use of PZQ raises concerns about potential drug resistance, especially with immature flukes. There is ongoing debate about the potential for PZQ resistance. Some studies suggest that variations in the host's CYP enzymes, rather than extensive PZQ use, might reduce the drug's effectiveness [7, 17, 18].

Novel drug delivery methods, including lipid‐based nanocarriers, are being explored to enhance PZQ's bioavailability and anti‐schistosomal activity [19]. Additionally, combining PZQ with other treatments like UDCA has shown promise in reducing worm burden and improving liver function in schistosomiasis patients. However, the potential development of PZQ resistance has led researchers to investigate alternative treatments like artemether [23].

The need for a schistosomiasis vaccine is urgent due to the limitations of PZQ. A vaccine, even if partially protective, could help reduce infections and hinder transmission. Targeting the early stage of the parasite's life cycle, specifically between cercarial skin penetration and schistosomula presence in the lungs, is seen as a promising approach. This stage is likely when the parasite is most vulnerable to immune attack, and targeting it avoids the immunopathological issues associated with eggs released by mature worms [37].

However, advances in immunology and systems biology offer opportunities to identify highly immunogenic proteins from schistosomes, which could be used in new vaccine formulations [46].

PZQ also has significant immunomodulatory effects, enhancing the immune response against schistosomes and potentially affecting the efficacy of vaccines. It has been observed to increase the recognition of schistosome antigens and enhance antibody responses, which may support the co‐administration of PZQ and vaccines. However, more research is needed to determine the optimal combination of PZQ and vaccines in treating schistosomiasis.

For a schistosomiasis vaccine to be more cost‐effective than the current treatment, PZQ‐based chemotherapy, it would need to provide long‐lasting protection and be integrated into the routine immunization schedule for children. However, cultural and behavioral factors, such as parental perceptions of vaccine efficacy, could influence the success of vaccination programs [47].

Morover the target population's previous exposure to schistosomes and differences in immune responses complicate vaccine trials. Schistosomes can cause immunological suppression, particularly in populations with persistent infections, which may affect vaccine outcomes [34].

Progress has been made in developing vaccines against *S. mansoni* and *S. japonicum*, with a focus on recombinant antigens and blocking transmission in animals. These vaccines need to be stable and effective in tropical regions where schistosomiasis is endemic. Experimental animal models are essential for testing vaccine efficacy and understanding disease progression, while antibody assays help evaluate vaccine effectiveness [48].

Initial human vaccine trials, like the one against *S. haematobium* (rSh28GST), have had poor results. However, other vaccine candidates, such as Sm‐TSP‐2, Sm‐p80, and Sm14, are in early‐phase studies. For *S. japonicum*, vaccination of cattle, which are major reservoirs, is also necessary as part of a “transmission‐blocking vaccine” strategy [38, 39].

The development of a schistosomiasis vaccine is focused on targeting various components of the *Schistosoma* parasite:

1. **LCP targeting Sm‐CatD**: The LCP system is a promising vaccine strategy that eliminates the need for additional adjuvants. It targets the cathepsin D hemoglobinase (Sm‐CatD) of *S. mansoni*, crucial for the parasite's digestion of blood. LCPs have shown effectiveness in generating antibodies that inhibit Sm‐CatD activity and bind to the parasite's tissues, suggesting their potential as a vaccine component [49].
2. **Schistosome membrane proteins**: The parasite's tegument, particularly its tetraspanin family of membrane proteins, is a critical target for vaccines. Proteomics studies have identified these proteins as promising candidates for inducing protective immunity, making them a key focus for vaccine development [50].
3. **PKs in *S. japonicum***: PKs play essential roles in the growth, development, and host interactions of *S. japonicum*, particularly in Asia. Targeting PKs offers new avenues for developing treatments and vaccines against schistosomiasis [51].
4. **SJ67 as a vaccine candidate for *S. japonicum***: The 67 kDa surface membrane protein (SJ67) has shown potential as a vaccine candidate, recognized by antibodies in schistosomiasis‐infected individuals. Recombinant SJ67 has been successfully expressed and purified, indicating its promise in vaccine development [52].
5. **Schistosome SEA as adjuvants**: SEA from *S. mansoni* have been found to enhance immune responses when used as adjuvants in vaccines, particularly in increasing Th1 and cytotoxic T cell responses. SEA, and specifically omega‐1, can modulate immune responses, potentially improving vaccine efficacy against other diseases, including HIV‐1 [53].

Despite their negative impact on vaccine efficacy, helminth infections in endemic areas may reduce the prevalence of allergic diseases, possibly due to changes in immune responses during pregnancy and early life. This complex interaction between helminth infections and immunity highlights the need for careful consideration in vaccine development and deployment in endemic regions [58].

Chronic helminth infections, such as schistosomiasis, can skew the immune system toward a Th2 bias, potentially reducing the effectiveness of vaccines against diseases like tuberculosis, malaria, and HIV‐1. Helminth infections can also suppress vaccine‐specific immune responses, especially in populations with maternal or early‐life exposure to these parasites Maizels and McSorley [55].

The development of a schistosomiasis vaccine is seen as a critical tool to complement MDA efforts and achieve significant improvements in disease control.

**Figure 4:**
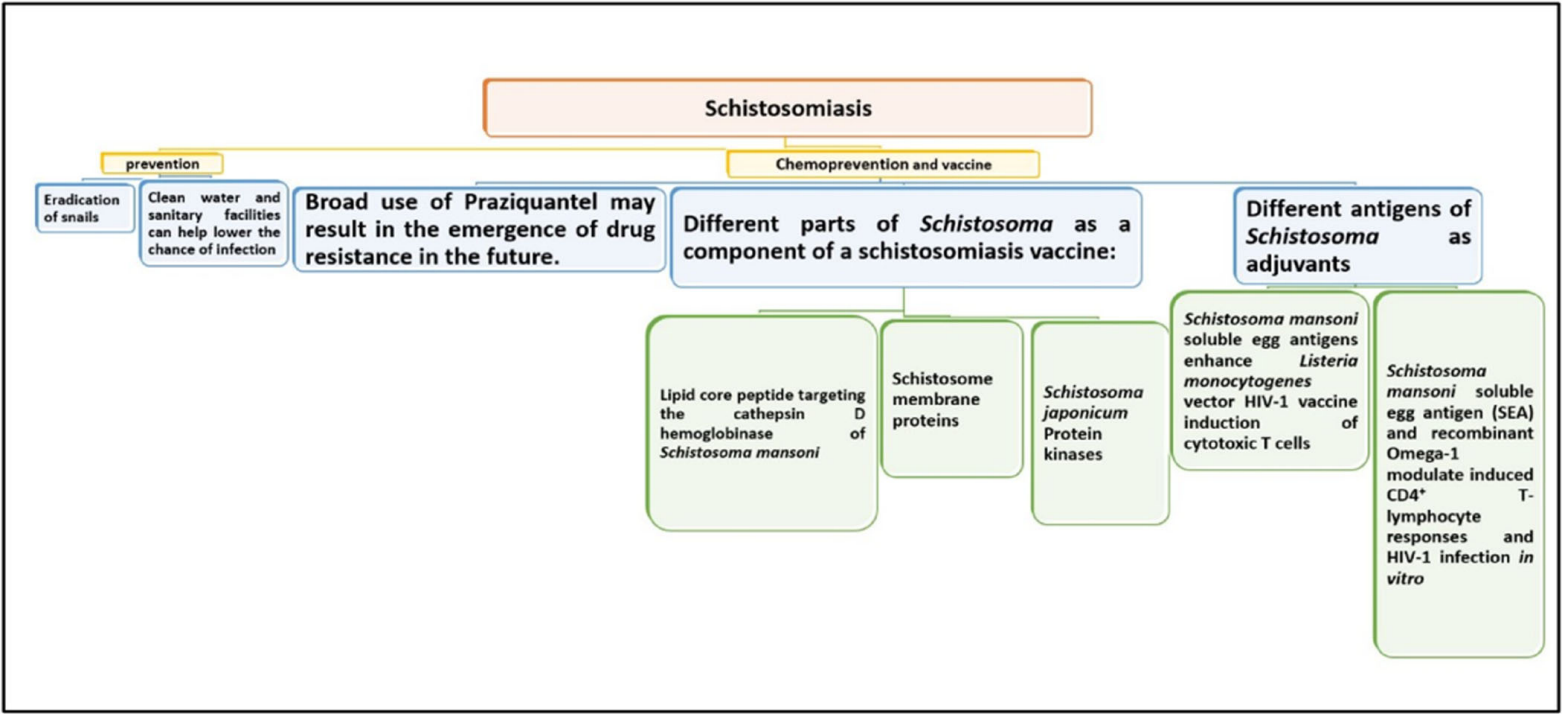
Summary of schistosomiasis prevention and control.

The WHO encourages continuous research and development of new tools and treatment options for schistosomiasis elimination. Vaccination is a critical component in the control of many illnesses. However, many decades of vaccine development for schistosomiasis have been less successful, with several promising preclinical candidates failing to reach human clinical trials. The pursuit for a schistosomiasis vaccine, on the other hand, is an extra kit to the repertoire of schistosome control measures. Indeed, 50 experts identified a schistosomiasis vaccine as one of the top 10 vaccines in need of urgent research (Figure 4) [37]. To advance schistosomiasis vaccine development, there is an urgent need for an effective vaccine pipeline that leverages cutting‐edge technologies like mRNA vaccines and CRISPR. Improved research tools and models are crucial for evaluating new approaches and enhancing vaccine design, production, and deployment [48].

## 5 Conclusion

Favorably PZQ is a medication with excellent chemopreventive treatment compliance. Due to the extensive usage of PZQ, there is a great deal of debate surrounding the emergence of drug resistance. PZQ is effective against all species of schistosomes, schistosomiasis prevalence has remained largely unaffected, due to reinfection in high transmission areas and growing juvenile worms that were not affected by the drug, even though the need for a schistosomiasis vaccine is made even more pressing. Different parts of *Schistosoma* can be used as a component of a schistosomiasis vaccine or as an adjuvant.

## Author Contributions

All authors contributed to the study's conception and design. Material preparation, data collection, and analysis were performed by Walaa A. Elkholy, Mona M. El‐Derbawy, and Noha F. Zahran. The first draft of the manuscript was written by Gaber El‐Saber Batiha, Athanasios Alexiou, Marios Papadakis, and Alaa Oqalaa E. Alibrahim help in rewriting, editing, and polishing. All authors commented on previous versions of the manuscript. All authors read and approved the final manuscript.

## Ethics Statement

This article is based on previously conducted studies and does not contain any new studies with human participants or animals performed by any of the authors.

## Conflicts of Interest

The authors declare no conflicts of interest.

## Data Availability

The data sets generated during and/or analyzed during the current study are available from the corresponding author on reasonable request.
